# Lifelong neurogenesis in the cerebral ganglion of the Chinese mud snail, *Cipangopaludina chinensis*

**DOI:** 10.1002/brb3.652

**Published:** 2017-03-03

**Authors:** Charles C. Swart, Amelia Wattenberger, Amy Hackett, Danielle Isaman

**Affiliations:** ^1^ Neuroscience ProgramTrinity CollegeHartfordCTUSA; ^2^ Trinity CollegeHartfordCTUSA

**Keywords:** Cerebral ganglia, *Cipangopaludina chinensis*, Gastropoda, Neurogenesis

## Abstract

**Introduction:**

A small group of Gastropods possessing giant neurons have long been used to study a wide variety of fundamental neurophysiological phenomena. However, the majority of gastropods do not have large neurons but instead have large numbers of small neurons and remain largely unstudied. We explored neuron size and rate of increase in neuron numbers in the Chinese mud snail, *Cipangopaludina chinensis*.

**Methods:**

Using histological sections and whole mounts of the cerebral ganglia, we collected cross‐sectional data on neuron number and size across the lifespan of this animal. Neurogenesis was verified using Click‐it EdU staining.

**Results:**

We found that total neuron number in the cerebral ganglia increases throughout the lifespan of this species at a constant rate. New neurons arise primarily near the nerve roots. Females live longer (up to 7 years) than males (up to 5 years) and thus achieve larger numbers of neurons in the cerebral ganglion. Neuron size is consistently small (<10 μm) in the cerebral ganglia at all ages, however, cells in the posterior section of the cerebral ganglia are modestly but significantly larger than cells at the anterior.

**Conclusions:**

These features suggest that *C. chinensis* and similar species of Caenogastropoda are good candidates for studying gastropod neurogenesis, senescence, and sex differences in the nervous system.

## 1 Introduction

The diversity of neuroanatomy, physiology, and behavior among the 50,000+ species of Gastropods provides a valuable resource for studying basic mechanisms of nervous system function (Chase, 2002). Indeed studies on several gastropod taxa have provided valuable inroads to studying wide ranging topics including learning and memory (*Aplysia californica*), plasticity (*Lymnaea stagnalis*,* A. californica*), and membrane receptor and channel function (*Conus* sp.) among others.

The large neuron size and fixed number of neurons in taxa such as *Lymnea* sp., *Aplysia* sp.*, Helisoma,* sp.*, and Helix* sp. makes them very attractive for studies of cellular physiology and neural network properties (Boyle, Cohen, Macagno, & Orbach, 1983; Bullock & Horridge, 1965; Chase, 2002; Dorsett, 1986; Gillette, 1991; Hermann, Watson, & Wildering, 2014; Longley, 2011; Zakharov, Hayes, Lerusalimsky, Nowakowski, & Balaban, 1998). However, the great majority of gastropods (especially those lacking giant neurons) remain unexamined beyond superficial anatomy.

This dichotomy of usefulness of the ‘giant but few’ neuron gastropods versus the unsuitability of the ‘many small’ neuron gastropods is well recognized (Boyle et al., 1983; Chase, 2002; Gillette, 1991). However, the former species have been invaluable for understanding the function of membrane channels, single cells or discrete neural networks, they may not be suitable for exploring adult neurogenesis, neuronal senescence and properties of large neural networks. The general knowledge derived from giant neuron gastropods has yielded a wealth of information to guide studies of vertebrates and, more relevantly, other little‐known gastropods (Gorbushin, Levakin, Panchina, & Panchin, 2001; Longley, 2011; Matsuo & Ito, 2011).

Plasticity in the nervous system can be achieved by modifying connectivity of individual neurons or multicellular networks OR by incorporation of new neurons into neural networks. The ‘Giant but few’ neuron gastropods seem to use the former mechanism as their primary means of plasticity increasing interneurons size and arborization to accommodate the new body tissue (Moffett, 1995). These gastropods are also thought to continually produce new sensory neurons in their peripheral tissue throughout their life (Moffett, 1995; Price, 1977). Little is known with certainty about adult neurogenesis in gastropods at the other end of the continuum (‘many small neurons’) except that adults do not have large or giant neurons (Boyle et al., 1983; Gillette, 1991; Lindsey & Tropepe, 2006). Plasticity in this large group of snails is likely due to lifelong neurogenesis (Gillette, 1991). Current research on adult neurogenesis in the vertebrate hippocampus is revealing how memory acquisition and storage involves both single cell plasticity and integration of new born neurons (Bergami et al., 2015; Johnston, Shtrahman, Parylak, Goncalves, & Gage, 2016).

Phylogenetically, the Gastropoda are divided into eight major clades of unresolved high‐order relationships (Bieler, 1992; Kocot, 2013). We studied the viviparid snail *Cipangopaludina* (*Bellamya*) *chinesis*, a large freshwater snail growing up to 60 mm long. It is native to East China, Taiwan, Korea, and Japan (Chiu, Chen, Lee, & Chen, 2002), but has been introduced accidently to North America and other countries around the world (Jokinen, 1983; Kipp, Benson, Larson, & Fusaro, 2014). The Viviparidae are placed within the Caenogastropoda well separated from the clade Heterobranchia that contains all species known to have ‘giant neurons’.

We were interested in understanding neuron proliferation in the cerebral ganglia across the lifespan in the Chinese mud snail, *C. chinensis*. Herein, we describe the anatomy of the cerebral ganglion and report the change in neuron number and size across the lifespan in both sexes. We find that *C. chinensis* exhibits neurogenesis across the lifespan at a steady rate in older juveniles, adult males and females. Females tend to live longer than males, reach adulthood at a larger size and attain larger body sizes and subsequently higher total neuron numbers. Neurogenesis in the cerebral ganglion is localized mainly near the base of peripheral nerve bundles.

## 2 Materials and Methods

*Cipangopaludina chinesis* snails were collected at two sites in Newington, CT, U.S.A.: Churchill Park Pond (Lat. Long. = 41.676, −72.721) and Mill Pond Park (41.693, −72.730).

The specimens were collected between April and October from 2009 to 2012. The species of all snails collected was identified using the key, Freshwater Snails of Connecticut (Jokinen, 1983). No animals with the greater length to width ratio indicative of the uncertain species or morphotype “*Cipangopaludina japanensis*” were present at these ponds although they are present in other ponds in the region.

Upon collection, the snails were maintained in lab aquaria kept at 20^+/‐^ 3°C on a 16:8 dark/light cycle. Before dissection, width and length (mm) were measured using a digital vernier caliper and mass (g) was measured using a digital scale. Opercular rings were counted under a dissecting microscope using a horizontal light source to determine the age in years (Chen & Soong, 2002; Llano, Ito, Fujinaga, & Nakao, 2004). The snails were classified into three classes—juvenile, male, or female. We verified distinctly male and female animals based on the previously reported gender system for this animal (Stanczykowska, Magnin, & Dumouchel, 1971). All males, including juveniles possess a canal in the right tentacle for delivery of sperm. A snail was classified as female if it carried eggs or developing juveniles. In the presence of testes, a sample of the sperm inside the testes was placed on a glass slide and evaluated under a compound microscope at 10×. A snail was classified as male if the testes contained motile sperm. A snail was classified as a juvenile if the developing testes contained static sperm or if no testes or eggs or embryos were present.

**Figure 1:**
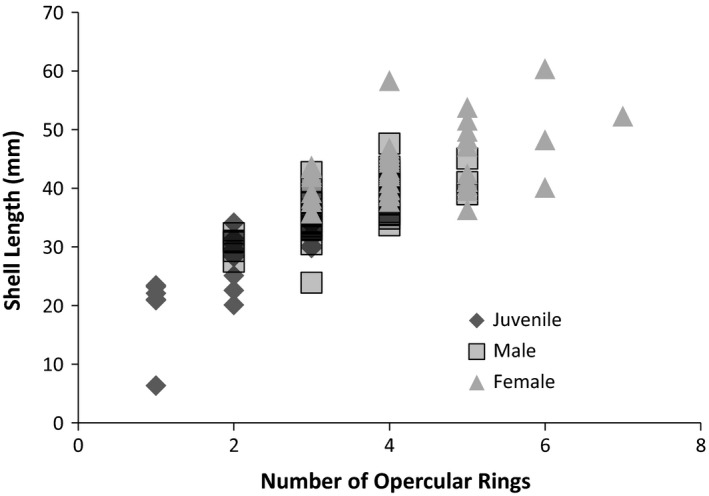
Relationship between body size and number of opercular rings. Previous studies with numerous gastropods have validated the use of opercular ring number with age

Cell counts were performed using several techniques. General distribution of neurons in the ganglion was observed using Paraffin embedded ganglia sectioned at 8 μm and stained with Hematoxylin and Eosin. Neuron counts were done on 46 whole mount ganglia stained with the toluidine blue stain described by Altman and Bell (1973). Stained ganglia were mounted on slides and observed under a compound microscope with a 10 × 10 mm grid in the eyepiece. To simplify the neuron counting system we used data from these 46 animals to validate a method for cell number estimation based on the surface area of the ganglion. Stained whole mount specimens had an average cell density of 20 cells per 0.005 mm^2^. This corresponds to the area on the slide enclosed by one square in a 10 mm × 10 mm ocular grid at a magnification of 200×. Neurons in the dorsal layer of the ganglia were present in a continuous, uninterrupted sheet whereas the ventral surface of the ganglion was divided into several populations of neurons separated by large axonal tracts (Figure 1). Thus, the number of neurons lining the ventral layer of the ganglion represented approximately 70% of the dorsal surface. The data on the density of neurons per surface area of the ganglia from the Toluidine blue stained specimens was used to estimate neuron number in all other specimens after dissecting the cerebral ganglia out and mounting them on slides in the same manner as the reference specimens. Surface area was determined by capturing a digital image of the specimen and measuring area using Image J.

The formula for computing the number of neurons in the dorsal layer of a ganglion was: Surface area (mm)  × 200 magnification × 20 neurons per 0.005^2^ mm. This value was multiplied by 1.70 to account for the neuron number in the ventral side of the ganglion. Note that this value must be multiplied by 2 to account for the bilateral ganglion. We analyzed data on the counts from a single ganglion. Overall measurements were taken from 26 juveniles, 46 adult males, and 52 adult females. We did not attempt to estimate the neurons along the edges of the ganglia, so our estimate is a slight underestimate.

Neuron size was also compared between regions of the cerebral ganglion from anterior to posterior. Ganglia from 30 animals (ten from each class) were examined under a compound microscope. An ocular micrometer, calibrated with a stage micrometer, was used to measure the diameter of the neurons in the regions of the ganglion as indicated in Figure 6. The diameter of 10 neurons in the dorsal surface and 10 neurons in the ventral surface were measured in each of the three regions of the brain (anterior, medial, posterior, Figure 6).

The cross‐sectional data supporting lifelong neurogenesis were verified using a Molecular Probes Click‐iT^®^ EdU Alexa Fluor^®^ 488 Imaging Kit on two adult males and two adult females. Experimental animals were anaesthetized by chilling on ice for 1 hr. An aperture was cut in the shell above the head using a rotary tool. Approximately, 20 μl of 10 μm Edu was injected into the body cavity adjacent to the cerebral ganglia using a 1 ml syringe with a 31 guage needle. Two animals, one male and one female were injected with 20 μl PBS and used as negative controls. Animals were held at room temperature for 48 hr before sacrifice and processing of the cerebral ganglia as described in the kit instructions. Images were obtained using a Retiga 2000R camera mounted on an Olympus BX‐41 epifluorescence microscope.

Statistical analysis of the change in neuron number with body size by class and basic descriptive statistics were calculated and graphed with Microsoft Excel. A MANCOVA on change in body size and neuron number across classes was conducted using Graphpad Prism 6.0.

## 3 Results

**Figure 2:**
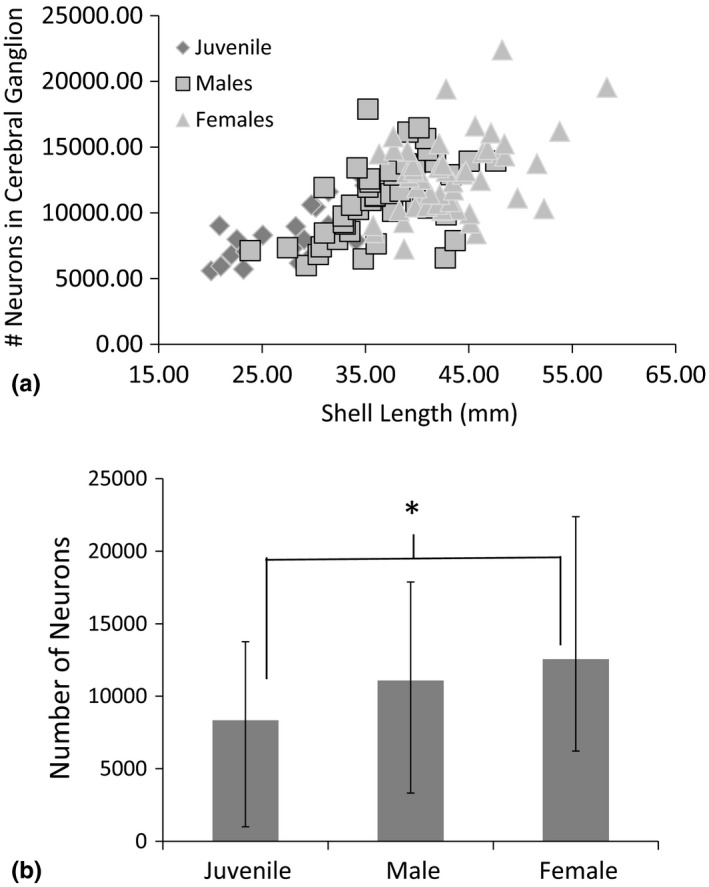
(a) Relationship between Shell Length and the number of neurons in one half of the paired cerebral ganglia in Juvenile, Male and Female *Cipangopaludina chinensis*. (b) Average and Range of neuron count in the cerebral ganglion of each class of *C*. *chinensis*. Note the low range for adult male and females are nearly equal while female's upper range extends well beyond males

Data for all animals were first analyzed to establish the relationship between opercular ring number and age (regardless of gender). The relationship between body size, age (i.e., opercular ring count) and neuron number was plotted (Figures 1 and 2) and analyzed using a regression analysis. Overall there is a positive relationship between Body size (shell length), age (opercular ring number), and neuron number. Body size increases 5.65 mm for each opercular ring (*R* ^2^ = .65, *F *= 226.9, *p *< .0001, *n *= 124) and neuron number increases by 1,835 cells with each ring (*R* ^2^ = .39, *F *= 77.7, *p* < .0001, *n *= 124).

**Figure 3:**
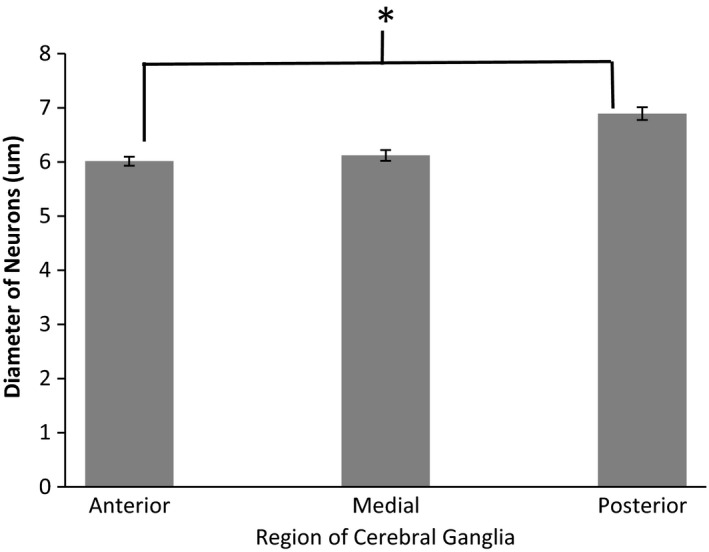
Average Diameter and Standard error of neurons in different regions of the cerebral ganglion of *Cipangopaludina chinensis* (10 cells in each of three brain regions in 10 animals of each class, See figure five for location of Anterior, Medial and Posterior)

Next, we examined the data using MANCOVA to determine the relationship between class (juvenile, male, female) and neuron number. Neuron number increased at a constant rate throughout the lifespan of *C*. *chinensis* snails, including juveniles and adult males and females (MANCOVA *p* = .83, Table 1). Neuron number in the cerebral ganglia increases as body size (shell length) increases (within and across each class) (MANCOVA, *p* = .04, Table 1, Figures 1 and 2). A comparison of neuron size across classes showed larger neurons in females but no difference between males and Juveniles (Table 2, ANOVA *p* < .01, Figure 2). When the cerebral ganglion is divided into anterior, medial, and posterior sections, we did find that cells generally increase in size from the front of the cerebral ganglion to the posterior part (Figure 3). Statistical comparison of the diameter of cells in the anterior and posterior portions of the ganglion indicate a statistically significant difference (*t *= 6.12, *p* < .0001). There was low heterogeneity in size of cells (despite statistical significance) compared to other gastropods.

**Table 1:** Results of a MANCOVA analysis examining the rate of increase in neuron number by size and the overall number of neurons between Juvenile, Adult Male, and Adult Female *Cipangopaludina chinensis*

	*F*	*df*	*N*	*p*
Rate of change in neuron number	0.181	2	101	.835
Number of neurons per class	3.310	2	101	.04

	Juvenile	Male	Female	
Slope	121.3 ± 16.57	141.1 ± 27.50	123.9 ± 23.94	

**Table 2:** ANOVA comparing diameter of neurons (μm) between juveniles, males and female *Cipangopaludina chinensis* (10 animals in each class, 18 cells total from each individual, six cells each from anterior, medial, and posterior cerebral ganglion)

Source	SS	*df*	MS	*F*	*p*
Treatment (between groups)	27.6764	2	13.8382	22.42	<.0001
Error	109.2427	177	0.6172		

**Figure 4:**
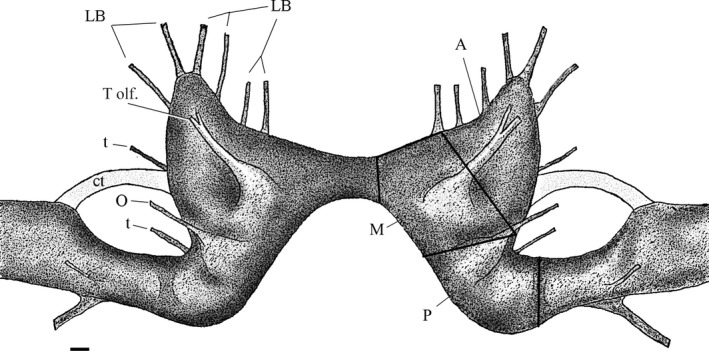
Cerebral ganglia of *Cipangopaludina chinensis*. Peripheral nerves from the cerebral ganglion are predominately involved in communication with the tentacles (T olf.  = Tentacular olfactory, O = optical, *t *= tentacular other) and the lips and mouth (LB = lips/Buccal cavity). Dark lines segmenting the Right ganglion indicate the three regions, we used to compare average size of neurons. Note the connective tissue (ct) joining the cerebral to the adjacent pleural ganglia. For the comparison of cell diameter, the ganglion was divided arbitrarily into Anterior (A), Medial (M), and Posterior (P) sections. Bar is approx. 0.1 mm

**Figure 5:**
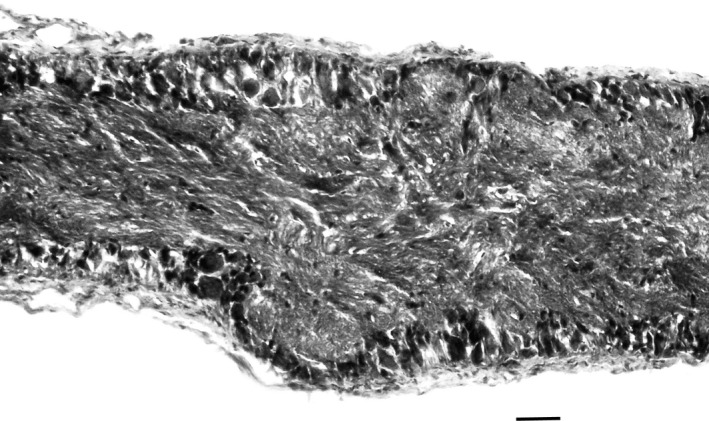
Cross‐section of paraffin embedded Hemotoxylin and Eosin stained cerebral ganglion of *C*. *chinensis*. Neurons are distributed along the inner surface of the membrane in layers of 1–3 cells. The middle of the ganglion is dominated by neuropile and occasional glia

**Figure 6:**
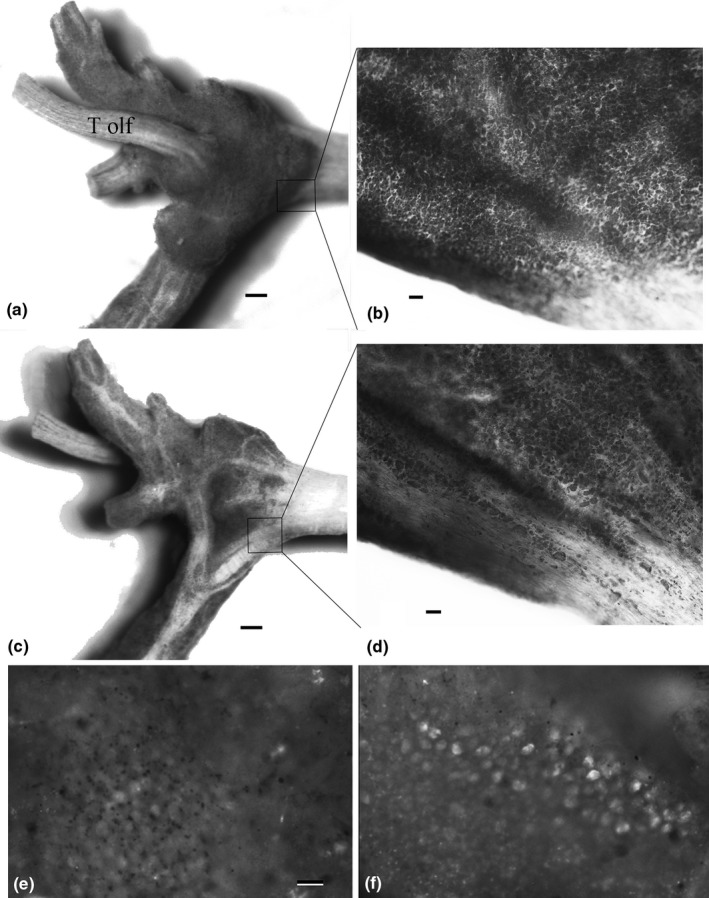
Whole mount of Left cerebral ganglion of *Cipangopaludina chinensis* demonstrating toluidine blue staining and EdU staining. (a and c) show the dorsal and ventral view of the ganglion after formaldehyde fixation, staining and removal of the neural sheath (T olf = Tentacular olfactory nerve). The dark areas are the neurons which have stained with the toluidine blue and the light areas are nerve fiber tracts (bar = approx. 0.1 mm.) (b and d) show the same specimen after dehydration, clearing in toluene, and mounting on a microscope slide (bar = approx. 20 μm). In (b), the focal plane is on the dorsal layer and in (d), the focal plane is on the ventral layer. Boxes are approximate area of the micrographs in (b and d). (e) Area around the base of the Tentacular nerve in a negative control animal injected with PBS instead of EdU showing background staining. (f) Base of the tentacular nerve on the cerebral ganglia of an Experimental animal injected with 20 μl 10 μM EdU for 48 hr (bar represents 10 μm)

The overall anatomy of the cerebral ganglion of *C. chinensis* is similar to that seen in typical gastropods and Viviparidae in particular. The symmetrical, bilateral cerebral ganglia innervate significant portions of the lips of the snail as well as the single paired chemosensory tentacles and the eyes (Figure 4). The pleural ganglia are located laterally with a very short commissure, thus exhibiting the ‘epiathroid’ condition (Chase, 2002). Within the ganglia, the cell bodies are concentrated proximally to the ‘epineurium’ with the central part of the ganglia filled with the axonal and dendritic extensions (the neuropile for local connectivity). Generally, the cells are distributed homogeneously in thin layers of 1–3 cells along the entire dorsal layer of the ganglia. Along the ventral layer, cells are similarly distributed across approximately 70% of the ganglia, and the remaining area is filled with large axonal tracts (Figures 5, 6). These ‘axonal tracts’ are distinctly different from the neuropile as the fibers are highly organized into parallel tracts as opposed to the apparent lack of organization in the neuropile, and are used for interganglionic connectivity (Chase, 2000).

## 4 Discussion

We found a constant rate of addition of cells to the cerebral ganglion as body size and age increases. This indicates that neurogenesis continues throughout life and that the total number of neurons increases throughout the lifespan; thus cell addition overcomes any possible cell senescence (if present). What is the role of neurogenesis in the adult brain of these snails? Three possible explanations are recognized in the literature; the replacement of naturally senescing older neurons, innervation of new body tissue as the animal grows and encoding experiences into memory. Research on Giant neuron snails, *L. stagnalis* and *Aplysia* sp. have documented changes in membrane excitability with age of critical giant neurons and the role of antioxidants in preserving the health of these critical cells (Hermann et al., 2014; Moroz & Kohn, 2010). Normal afferent and efferent innervation of new body tissue during adult growth has received very little attention in either vertebrates or invertebrates; probably because it is considered to be simply a continuation of the processes that occur rapidly during early development. Memory formation and storage, along with cell senescence is one of the largest fields of inquiry in modern neuroscience. The gastropod *Aplysia* was and is key models for understanding the molecular mechanisms of memory formation and storage. Numerous studies have documented rudimentary abilities of gastropods (primarily Giant Neuron gastropods) to form memories through habituation, classical conditioning, and aversive conditioning (Hermann et al., 2014). Senescence, normal innervation of new tissues, and memory storage in snails with lifelong neurogenesis, such as *C*. *chinenesis* remains unexplored but may offer interesting contrasts to Giant neuron senescence and cell aging/neurogenesis in the vertebrate hippocampus.

The number and size of neurons in gastropods is said to range from animals with few but very large cells on one end of the continuum and animals with many small cells on the other. *C*. *chinenesis* falls in the latter (Boyle et al., 1983; Gillette, 1991). Adult male *C*. *chinensis* range from 3,000 to 9,000 neurons in one of the paired cerebral ganglia and females range from 6,000 to 20,000 cells (increasing with age/size). This is in good agreement with the range of neuron counts seen in other Caenogastropoda (Boyle et al., 1983). Boyle et al. (1983) presented data on total numbers of neurons in the entire nervous system of a variety of snails and slugs. Large neuron species have cells ranging in diameter from approximately 20 μm up to 1 mm with the majority of cells in the 25–100 μm range. Generally ‘giant neuron’ snails have only few (between 3 and 11) truly giant neurons (>0.5 mm). Total numbers of cells for all ganglia ranged from 4000 in the sea slug, *Navanax inermis,* up to approximately 50,000 in the freshwater ‘pulmonate’ snail, *L. stagnalis*. With up to 20,000 cells in one of the paired cerebral ganglia in *Cipangopaludina* the total number of cells in the nervous system likely exceeds 200,000 cells in large animals (if surface area of the ganglia is an accurate predictor).

The juvenile period lasts 2–3 years before animals become sexually mature. Males have an overall lifespan of 4–5 years while females typically live up to 7 years. Females mature later than males; they remain juveniles until attaining a larger size. The size at maturity seems to be more strictly determined for females. This may agree with predictions from the point of view of a cost/profit curve for energy investment in zygotes (Blanckenhorn, 2000). Because body size and neuron number increases continually, females of this species achieve larger body size as well as neuron number. The increased time to maturity in females affords a potentially valuable time window for discovering parts of the brain in this animal that are related specifically to female reproduction and behavior. Cells that arise in areas of the cerebral or other ganglia in the third year of females would likely be related to gender differences. Previous work has shown the presence of reproduction related peptides in the cerebral ganglion of several gastropods (Ram, Gallardo, Ram, & Croll, 1998).

We do show a gradient of increased size in neurons from the anterior to the posterior limit of the cerebral ganglion; but we do not have definite evidence of increase in size of individual neurons across a lifespan. The size increase we show from anterior to posterior of the cerebral ganglion likely reflects age of the neurons, as it is similar to the evidence reflecting this phenomenon derived from studies of *Helix aspersa* (Longley, 2011) and *Helix lucorum* (Zakharov et al., 1998). A similar proliferative region on one particular part of the membrane of the abdominal ganglion has been shown in *Aplysia* (Hickmott & Carew, 1991). There is other evidence that neural precursors for gastropods arise in the ectoderm and invade the central nervous system through major nerves (Hickmott & Carew, 1991; Jacob, 1984; Longley, 2011). However Neural precursors in crayfish (*Procambarus clarki*) are now thought to enter the brain from the hemocoel (Zhang, Allodi, Sandeman, & Beltz, 2009). The anterior region of the gastropod cerebral ganglia may be a productive area to further explore the origin of neural precursors. The EdU proliferation assay showed fairly robust staining after 48 hr indicating a significant rate of neurogenesis. The cellular and molecular biology of the origin and differentiation of stem cells in snails is largely unexplored.

While lifelong neurogenesis and neural regeneration in response to tissue damage are clearly different processes, the possession of the former characteristic seems to be vital for the ability to perform the latter (Moffett, 1995). The marine pulmonate snail, *Melampus* is known to regenerate the entire procerebrum as well as the tentacle and eventually normal behavior after ablation (Moffett & Ridgway, 1988; Moffett & Snyder, 1985; Price, 1977). The prosobranch and ‘small neuron’ snail *Hydrobia ulvae* can regenerate buccal and parapodial ganglia along with other head structures after ablation (Gorbushin et al., 2001). Many studies have reported on the regrowth of sensory structures in snails (Eakin & Ferlatte, 1973; *Helix aspersa* eye; Chase & Kamil, 1983; *Achatina fulica,* tentacle; Flores, Brusco, Scicolone, & Saavedra, 1992; *Cryptomphalus (Helix) aspersa,* eye), and exhibit regeneration of neural tissue after injury (Melampus sp *H. lucorum* (Zakharov et al., 1998), *Helix aspersa* (Eakin & Ferlatte, 1973), *Crypomphalus aspersa* (Flores et al., 1992), *Limax valentianus* (Matsuo & Ito, 2011), and *Limax* sp. (Watanabe, Kirino, & Gelperin, 2008). Neurogenesis during early development has been described for several species including *Helix aspera* (Longley, 2011), *L. stagnalis* (Croll & Voronezhskaya, 1996), *Aplysia californica* (Jacob, 1984), and *H. lucorum* (Zakharov et al., 1998) all species with giant neurons. However, several studies have reported adult neurogenesis in ‘large neuron’ gastropods, particularly in the Terrestrial Pulmonate slugs. These species exhibit adult neurogenesis as well as remarkable recovery of neural structures after damage. Ratté and Chase (1997) have shown that the terrestrial snail *Helix aspersa* exhibits significant neurogenesis as an adult, particularly in the procerebral region of the cerebral ganglia.

Modern instrumentation and advancements in genetic, cellular, and molecular techniques now make it possible to study the complex phenomena of learning and memory, senescence, and behavior in vertebrates. However, nonvertebrate model organisms still have much to offer particularly diverse phyla where several alternative genetic/cellular/molecular mechanisms have arisen for generating these phenomena.

## Conflict of Interest

None declared.
